# Improving Treatment Adherence and Retention of HIV-Positive Women Through Behavioral Change Interventions Aimed at Their Male Partners: Protocol for a Prospective, Controlled Before-and-After Study

**DOI:** 10.2196/19384

**Published:** 2021-01-25

**Authors:** Stefano Orlando, Ilaria Palla, Fausto Ciccacci, Isotta Triulzi, Darlington Thole, Hawa Mamary Sangaré, Maria Cristina Marazzi, Karin Nielsen-Saines, Giuseppe Turchetti, Leonardo Palombi

**Affiliations:** 1 Department of Biomedicine University of Tor Vergata Rome Italy; 2 Institute of Management Scuola Superiore Sant’Anna PIsa Italy; 3 Unicamillus Saint Camillus International University of Health Sciences Rome Italy; 4 DREAM programme Community of Sant'Egidio Balaka Malawi; 5 Libera Università degli Studi Maria Ss Assunta di Roma Rome Italy; 6 David Geffen UCLA School of Medicine University of California, Los Angeles Los Angeles, CA United States

**Keywords:** retention in care, therapeutic adherence and compliance, men's role, acquired immunodeficiency syndrome, AIDS, HIV, behavior, intervention study, health education, community health education, Malawi, mother-to-child transmission, health-related behavior, social ecology

## Abstract

**Background:**

According to the World Health Organization, in 2018, 37.9 million people were living with HIV globally. More than two-thirds were residing in sub-Saharan Africa, where the HIV prevalence in the adult population (aged 15-49 years) was 3.9%. This population included 1.3 million pregnant women, of whom 82% had received antiretroviral therapy (ART) for the prevention of HIV mother-to-child transmission. In these countries, one challenge is an insufficient level of treatment adherence, particularly in HIV-positive pregnant women. Among the causes, the lack of involvement from a male partner is a significant contributor to the problem. This issue has strongly emerged in Malawi, one of the countries with the highest HIV prevalence in the world: 9.2% of its adult population were living with HIV in 2018.

**Objective:**

This study aims to assess 3 interventions that are aimed at improving ART adherence and retention among HIV-positive women through engagement with their male partners in 4 Malawian health care centers.

**Methods:**

The prospective, controlled before-and-after study is conducted in 3 phases (total duration: 24 months): preintervention, intervention, and postintervention analyses. The number of selected clusters (clinical centers) is limited to 4: one for each intervention, plus a cluster where no intervention is performed (control arm). The interventions are as follows: opening the facility on one Saturday per month only for men, defined as a *special day*; testing peer-to-peer counseling among men, *male champions*; and providing a noneconomic incentive to all women who are accompanied by their partners to the facility, *nudge*. The primary outcome of the study is to evaluate the differences in retention in care and adherence to therapeutic protocols among women; the intermediate outcome is the assessment of differences in male involvement. The level of male involvement in the health of their partners (intermediate outcome) will be evaluated through a dedicated questionnaire administered at baseline and in the postintervention phase. Data will be collected at the clinical centers and stored in 2 electronic databases managed using 2 different types of software.

**Results:**

The analysis of data collected in the 4 centers during the preintervention phase is ongoing, as enrollment ended on March 31, 2020. The total number of patients enrolled was 452 (Namandanje: 133; Kapeni: 78; Kapire: 75; and Balaka: 166). Meanwhile, several meetings have been conducted to organize the intervention phase.

**Conclusions:**

The study will identify the best intervention that enhances the involvement of male partners in women’s health, using an approach that considers a broad spectrum of behaviors. An important aspect is the use of educational tools focused on messages, thereby initiating a reflective discussion of stereotypes and false beliefs related to the idea of masculinity present in the Malawian culture.

**International Registered Report Identifier (IRRID):**

DERR1-10.2196/19384

## Introduction

### Background

According to the World Health Organization, in 2018, 37.9 million people were living with HIV globally [1]. The impact of this disease varies considerably across countries and regions. More than two-thirds of people living with HIV are in sub-Saharan Africa (SSA), where HIV prevalence in the adult population is 3.9%. Globally, there were 1.3 million pregnant women living with HIV in 2018 in the region, of whom 82% had received antiretroviral therapy (ART) for the prevention of HIV mother-to-child transmission (PMTCT).

In 2014, the Joint United Nations Programme on HIV/AIDS launched the *90-90-90* program. According to this ambitious plan, 90% of all people living with HIV would know their HIV status, 90% of all HIV people would receive ART, and 90% of all people receiving ART would have viral suppression by 2020 [2]. In most SSA countries, however, the level of adherence is suboptimal, particularly in HIV-positive pregnant women, among whom the adherence rate varies considerably across different settings, both within and across countries, ranging from 35% to 93.5% [3]. There are several barriers to medication adherence that are often linked to different individual factors (sociodemographic and knowledge base), therapy-related factors (complexity of therapy and side effects), social and economic factors, and health care provider and health system–related factors. Stigma and discrimination at the community level and the fear of disclosure of one’s HIV status to their partner are more significant barriers than others [4]. In this scenario, a critical issue is represented by the lack of male partner involvement along the continuum of HIV health care services. This issue represents one of the main reasons for treatment refusal, delayed enrollment, dropout, and low retention of pregnant and breastfeeding women [5-8]. The partner’s lack of involvement, on the contrary, is due to cultural, societal, and gender factors, socioeconomic factors, health service barriers, and policy gaps [9]. Barriers operate at different levels: community (gender issues, role on reproductive health, and stigma), health system (health services tailored to women’s needs and negative attitude of professionals), interpersonal (discordant couples and disclosure issues), and individual (scarce maternal knowledge of child health and fear of stigma) [9]. Therefore, an important challenge is to attain the support of male partners in promoting the health of their female partners. This process includes attending clinics, testing for HIV, and treatment adherence.

Studies have demonstrated that many countries have highly gender-specific and women-centered sexual and reproductive health programs and services [10]. Therefore, men see health-seeking in a maternal setting to be a *woman’s task* and the antenatal clinics as a space for women, and believe that the activities performed are outside their area of responsibility [11]. They perceive that visiting the antenatal clinic would be *unmanly* and they fear stigmatization by other men [11]. The norms related to masculinity discourage men from acceding to health facilities, in particular, to participate in antenatal care (ANC) [12].

Although the importance of male involvement and potential barriers to male involvement are well known, it is not yet clear which public health interventions are effective for attaining male involvement in the health of their female partners, resulting in positive health outcomes for women. There is currently a lack of high-quality studies evaluating the effect of male involvement on women’s health [9]. A 2012 Cochrane review identified only one eligible study pertaining to this issue [13], and a more recent review identified only 12 studies, 6 of which were observational [14]. A limitation of the studies conducted so far on the topic is that they are generally not framed in a theory, model, or framework. Only one of the aforementioned studies designed interventions based on a theoretical model [15]; however, the theory was not considered in the evaluation of results. The use of a theoretical framework allows researchers and health care professionals to be able to design and evaluate interventions by analyzing elements that may favor or decrease the effectiveness of each intervention. This improves the potential for reproducibility of the most effective interventions in different contexts (also known as external validity) [16].

### Design of the Study and Theoretical Framework

In this way, a prospective controlled study is more suitable for the evaluation of promising interventions to improve male involvement and enhance maternal adherence to HIV PMTCT.

For this study, we adopted a framework based on the ecological model [17] adapted by Kaufman et al [18] to health behaviors in HIV prevention. This model considers not only the PMTCT programs that HIV-positive women are engaged with but also the way they live and their relationship with their families and communities. According to the ecological model of health behavior, these are influenced by someone’s living environment, and this influence is articulated at multiple levels. This model, unlike models such as the Health Belief Model [19], does not focus only on individuals and their choices but tries to intervene on all external causes that support or limit positive individual actions [20-22]. In our study, this model aims to overcome the limitations of various adherence support programs that are exclusively based on health education and awareness-raising activities. In many cases, even though HIV-positive women know the importance of adhering to their ART regimens, external elements are preventing them from complying with their prescriptions. These include the attitude of their partner [23], the stigmatization by their community, and the lack of needed resources, both physical and mental.

**Figure 1:**
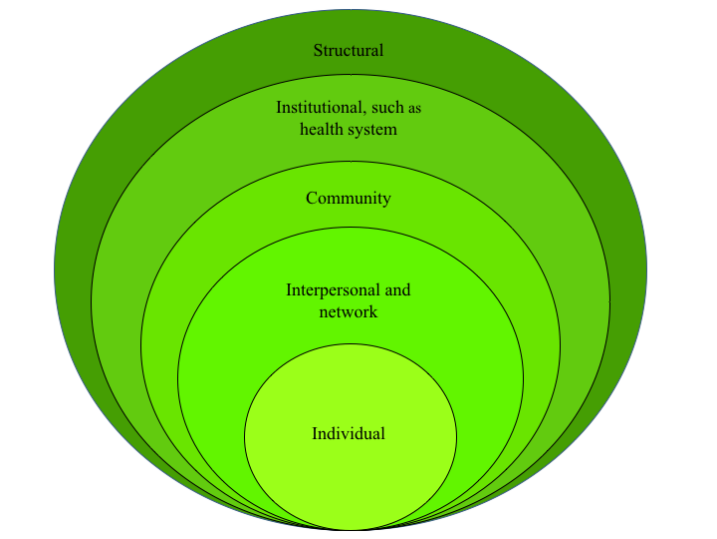
Effect of multiple integrated component interventions on the social ecological model.

According to the ecological model, many factors affect male involvement in women’s health care processes at different levels. In this study, we focused on the level presented in Figure 1, namely, individual level, interpersonal and network (eg, family and peers), community, health system (health facility level), and structural level (eg, access to transportation). These critical issues have strongly emerged in Malawi, one of the countries with the highest prevalence of HIV in the world: 9.2% of the adult population (aged 15-49 years) were living with HIV in 2018, and 59.8% of them were women. In 2018, 1 million Malawians were living with HIV, and 13,000 Malawians died from AIDS-related illnesses in the same year [24]. In July 2011, Malawi became the first country to implement the Option B+ approach, which means that all pregnant women living with HIV are offered antiretroviral treatment for life, irrespective of their CD4 count [25]. Between 2011 and 2018, the proportion of female adults with HIV who were diagnosed ranged from 49% to 94% [26]; the proportion of pregnant women with HIV who were virally suppressed jumped from 2% to 48%. The impact of this program has been huge, with a drastic reduction in HIV mother-to-child transmission (MTCT) rates; however, the percentage of male involvement in MTCT and maternal-child health services is still considerably low, ranging from 3.2% to 23% [27].

### Aim of the Study

This study aims to evaluate 3 interventions focused on different levels, as described in the ecological model. The primary outcome will be retention and adherence in the care of HIV-positive women, whereas the secondary outcome will be the involvement of male partners in the care of their partners.

## Methods

### Strategy of the Study

The objective of the study is to evaluate 3 different interventions aimed at improving adherence and retention to ART therapy among HIV-positive women through engagement with their life partner. The interventions are (1) testing peer-to-peer counseling and community-level health education sessions delivered by men, *male champions*; (2) opening the facility once a month, only for men, *special day*; and (3) providing incentive to all women accompanied by their partners at the facility, *nudge* or *incentive*.

The different interventions to be implemented and evaluated in the research plan are designed to assess male involvement at the following levels, as depicted in the model represented in Figure 1. The activities that act on the individual and interpersonal or at the social network level common to past studies are transversal to all 3 interventions. At the individual level, both women and their male partners will receive educational content on the importance of retention and adherence to treatments, but also messages that contradict stereotypes and false myths related to masculinity, health, and gender violence. With regard to the interpersonal or social network level, all interventions will include support activities that are aimed at the family (couples counseling).

- *Intervention 1* (*Male Champions*) will act at the community level, providing educational interventions that are aimed at territorial communities and religious groups.
- *Intervention 2* (*Special Day*) will act at the health system level, delivering activities based on health facilities that are aimed at reshaping the clinic as male-friendly.
- *Intervention 3* (*Nudge*) will also act at the structural level by providing incentives to support the cost of transportation and other costs that reduce a male partner’s ability to access the service.

**Figure 2:**
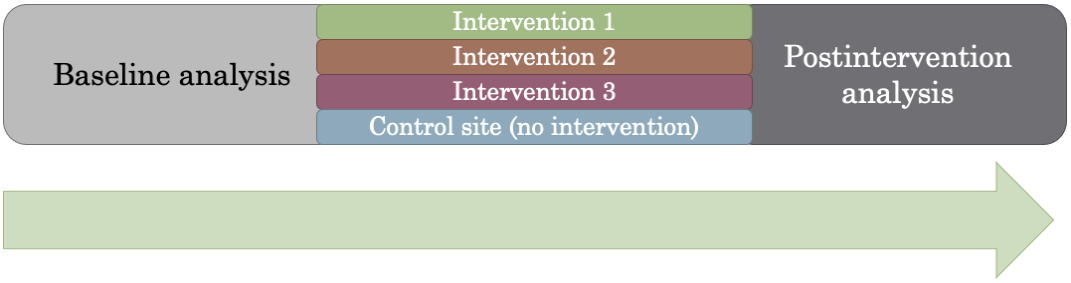
General structure of the study.

The study has 3 phases: preintervention (baseline), intervention, and postintervention analyses, as shown in Figure 2.

In the preintervention analysis, the baseline situation of the 3 participating centers will be evaluated. The aim of this phase is to set a starting point to weigh the relative effect of each intervention to be studied. For that purpose, baseline data of each study site will be collected. To evaluate the effect of the interventions, the same indicators will be measured during the pre- and postintervention phases, as described above. The preintervention phase will last 9 months. During this period, data will be collected at each site and included in a database.

### Setting and Participants

The study was performed in Malawi within the Disease Relief through Excellent and Advanced Means (DREAM) program. DREAM is a health program managed by the Community of Sant’Egidio in 11 African countries [28]. The program delivers a number of health services: HIV care, HIV PMTCT, malnutrition prevention and control, care of noncommunicable diseases, tuberculosis diagnosis and treatment, cervical cancer screening, and treatment of early lesions [29-33]. The DREAM program in Malawi runs 13 health centers and 3 laboratories in collaboration with the Ministry of Health. Currently, the DREAM program in Malawi has cared for over 86,000 patients and assisted over 12,000 HIV-positive pregnant women in the prenatal care and delivery of HIV-uninfected babies. In addition, the DREAM program aims to address health inequalities related to HIV, along with barriers experienced by women in accessing services. For this purpose, an expert client model aimed at improving retention in care and facilitating the inclusion of social aspects in the clinical process was launched [34]. However, activities involving male partners with the goal of improving access of their partners to services have never been tested in this model.

Given the limited resources available for the study and the practical difficulties related to the disadvantaged context in which the study was conducted, sample size calculations and criteria for assigning interventions were based on a pragmatic approach. The number of selected clusters is limited to 4, one for each intervention plus a cluster where no intervention will be performed as a control arm. The number of participants enrolled for data collection is given by the number of patients attending the selected clinical centers during the pre- and postintervention phases. Therefore, the main unit of analysis of the study is the cluster, which is the clinical center. Thus, the interventions will target each health facility.

The following criteria were followed to select the 4 centers included in the intervention:

1. The centers represented the Malawian population. As Malawi is a country where 83% of the population resides in rural or semirural environments [35], rural clinics were selected.
2. Centers that only provided maternal services were excluded because it was not possible to use a male-friendly approach in a center that is designed to accommodate only women.
3. The remaining centers with the highest number of female patients were selected.

The *special day* intervention was assigned to the center of Balaka (Balaka district). At this site, health care staff are available to offer the planned services. The *male champion* intervention was assigned to the Kapire center (Machinga district), where there are patients available to engage themselves as male champions who have sufficient levels of education necessary to perform the task assigned to them and communicate with the researchers. The *nudge* intervention was assigned to the Kapeni center (Blantyre district), where the number of women being treated was consistent with the available budget necessary for the provision of noneconomic incentives.

Although the unit of analysis is the clinical center, and therefore all patients who are referred, the study focuses on the population of adult females and the influence of male partners on the health practices of women. Therefore, data will be collected from this specific population. Eligibility criteria for women included the following: being HIV positive; inclusion in an HIV/AIDS prevention and treatment program; aged 18 years or older; and living at home with a male partner.

There are several possible criteria for defining a partnership relationship. In the Malawian context, the concept of marriage is understood in many different ways depending on local tradition or different religious practices [36]. Furthermore, mobility is quite high and there are situations in which the married partner spends longer periods away from the family or abroad; therefore, there is no possibility of directly influencing his wife's health practices. Hence, in this study, it was decided to consider the man who resides in the same house with the woman as a partner, regardless of religious or legal bonds between the two.

The enrollment of participants will be consecutively based on the order of patient appointments. Recruitment will be entrusted to doctors and clinical officers responsible for the medical visits of women participating in HIV treatment programs, who interact with patients during every visit to the clinical center. All women who met the eligibility criteria and agreed to participate in the study will be enrolled. Recruitment will be conducted before the medical examination: the software used for patient management reports to the clinician whether patients aged less than 18 years are enrolled, allowing the clinician to check entry criteria and invite eligible participants for study participation with the provision of informed consent.

### Data Collection and Storage

Data will be collected at the clinical centers and will be stored in 2 electronic databases managed through 2 different types of software:

1. Clinical and medical service data are routinely collected through an electronic DREAM program health record software used routinely at the clinical centers named DREAM_S [37].
2. Data on health behaviors reported by patients who participate in the study will be collected through the software created specifically for the study, also respecting rules for patient privacy and data security.

In the preintervention phase, data will be collected at the medical visit immediately after the enrollment visit to evaluate the partner's acceptance of the invitation to come to the clinical center for testing and counseling in the company of the patient. In the postintervention phase, data will be collected 2 months from enrollment to allow for a wash-out period where the effects of the interventions would be expressed.

### Interventions

There are several strategies and/or interventions to improve male involvement in the PMTCT process described in the literature. These include psychosocial approaches, verbal encouragement, invitation letters, community education, and sensitization. A review performed by Takah et al [38] showed that psychosocial approaches and complex community interventions are more effective, whereas invitation letters are ineffective. Our review of the literature showed that home visits are one of the most effective single interventions, whereas multicomponent approaches could improve male involvement [14]. After a meticulous analysis of the literature on possible interventions, 3 different types of interventions were selected: (1) community sensitization through male champions; (2) special day for a male-friendly clinic; and (3) incentive to attend the clinical center addressing structural barriers. Each intervention will be performed in a selected center (Table 1).

**Table 1:** The interventions.

Centers	Intervention	Ecological model levels	Activities
Kapire	Male Champions	Individual Interpersonal or social network Community	Individual counseling for men attending DREAM^a^ centers Home care for defaulting male patients Sensitization campaigns in local communities Sensitization campaigns in public spaces (churches, markets, clubs, and bars)
Balaka	Special Day	Individual Interpersonal or social network Health system	Basic health services: HIV testing and counseling Blood pressure measurement Counseling regarding cardiovascular disease Blood glucose measurement and counseling for diabetes Nutritional evaluation and counseling Group educational activities 2 educational sessions using the drama technique
Kapeni	Nudge	Individual Interpersonal or social network Structural	First visit Woman with partner: Food integration is given and an invitation card is delivered. If partners come for the second visit, they receive a second food integration Food integration is not given, but an invitation card is delivered, and if the male partner comes to the second visit, then the family receives a food pack Woman without a partner: Delivery food integration and registration that she has no partner Second visit with partner If the partner accompanies the woman to the center, the personnel will give a food package and offer couples counseling. The man can take advantage of individual services as educational contents

^a^DREAM: Disease Relief through Excellent and Advanced Means.

These strategies will be implemented according to standard clinical practices in Malawi with some modifications based on the international experiences described in the literature.

1. The *male champion* is a figure present in a few settings that conducts home visits with men and couples and follows up with men who did not accompany their partners to ANC visits [39]. Usually, this role is covered by the female expert clients, namely HIV-positive patients working in the organization as volunteers after appropriate training. Some men among patients who are receiving treatment will be identified and trained to cover this role [40]. The intervention called *male champion* aims to change the beliefs and attitudes of men, not intervening directly on individuals but upon entire communities and particularly involving community leaders.
2. The *special day* is a practice that is becoming increasingly common to facilitate access to health facilities by vulnerable groups such as adolescents [41,42]. In this study, we target men. The special day takes place with an extraordinary opening of the clinical center once a month on a prefestive day (Saturday). During this day, access will be reserved exclusively for men, including those who are not on treatment for HIV. The center will offer free health promotion services to all participants. This intervention aims to remove the attitude linked to beliefs regarding masculinity, in which the male partners see access to health centers as an activity suitable only for women. The special day aims to make the clinical center more male-friendly and therefore attempts to overcome the social pressure that pushes men to have only sporadic attendance.
3. The intervention based on the use of incentives (*nudge*) [43] to men who follow the prevention and treatment program aims to evaluate whether this action is effective in guiding behavior change toward the test, treatment, involvement, and adherence to therapy approach. The intervention defined as *budget* aims to remove the obstacles of a structural or a material nature, which supports the costs related to transportation to health care facilities and time taken away from work through a noneconomic incentive.

The educational tools developed for these interventions are based on standards adopted in the country for couples counseling on HIV/AIDS. To these standard guidelines, messages were added to initiate a reflective discussion of stereotypes and false beliefs related to the idea of masculinity in the Malawian culture. All staff involved in the delivery of these interventions were provided with a guide (included in Multimedia Appendix 1) on the contents to be delivered. They also participated in a one-day training of the themes of the project. The guide was developed by study investigators and Malawian health care staff. The guide was based on tools developed and applied in other contexts with the purpose of increasing men's access to health care services and to reduce stereotypes and gender-based violence. It was also based on the ecological model [44-49].

The educational content will be delivered using 3 different approaches. The *special day* arm will address the community in the catchment area of the clinical center, but the intervention will take place at the facility level. Through visibility activities such as leaflet distributions or information campaigns with audio messages, men living in the communities surrounding the treatment center will be invited to an extraordinary opening day at the center dedicated only to men. During the special day, the center’s clinical staff will offer basic health care services such as HIV testing and counseling, blood pressure measurement and counseling on cardiovascular disease, blood glucose measurement and counseling on diabetes, and nutritional evaluation and counseling. During the morning, group educational activities led by male community health care workers together with the doctors of the center and 2 educational sessions using drama techniques will be conducted. In the *male champions* arm, the intervention will be conducted at the level of regional and religious communities, with group educational sessions and use of the *male champions*. Male champions will be selected from patients receiving treatment for HIV for more than a year at the local DREAM centers. The educational sessions, based on an interactive approach with questions and answers both from the audience and the male champions, will last between 30 min to an hour. The male champion will be allowed to prolong the intervention if the audience requests it. Meetings will be planned and implemented in communities where at least five patients who receive treatment at DREAM centers reside, including religious communities in those regions. In the *nudge* arm, the intervention will be directed to individuals or to male partners of female patients being treated at DREAM centers. Women who agree to participate in the study will be asked to invite their male partners to the center for a health education session, and male partners who agree to participate will receive an incentive in the form of a food package, approximately worth US $8. At the second visit, the couple will be given a health education session based on educational materials prepared for the study.

**Figure 3:**
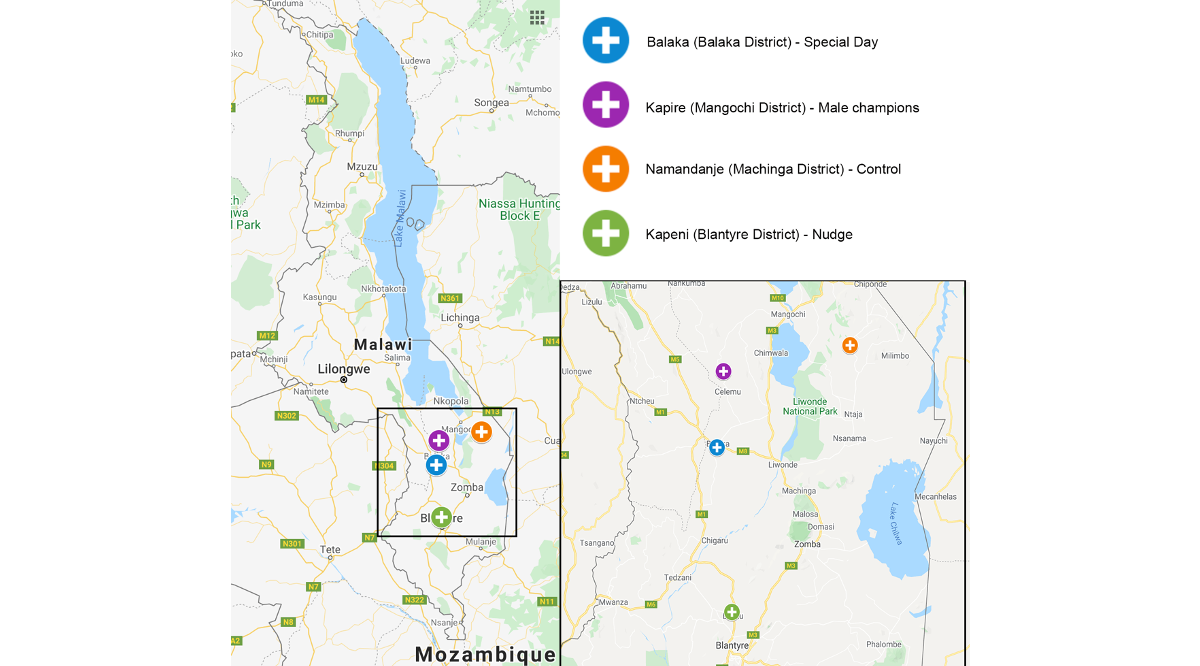
Geographical location of the 4 clusters.

The geographical locations of the 4 centers are shown in Figure 3. The centers are located at a distance that was considered sufficient to avoid geographical contamination. The 2 closest centers, Kapire and Balaka, are located at a distance of about 50 km; therefore, it can be reasonably excluded that patients can participate in both interventions. In any case, patients registered in the DREAM program and their family members are merged into a single database; therefore, the duplication of registration can be excluded.

### Outcomes and Statistical Methods

The outcomes will be divided into 2 levels: (1) evaluation of the variations in retention in care and women's adherence to therapeutic protocols (primary outcome) and (2) assessment of the variations in male involvement (intermediate outcome).

Retention in care and adherence to treatment protocols are primary outcomes. Retention in care will be measured as the cumulative proportion of dropouts from the treatment program at 6 months, 1 year, and 2 years (excluding patients transferred to another center or those who died). Adherence will be measured through the following indicators: (1) the cumulative proportion of medical appointments missed in a month in the center; (2) the cumulative proportion of appointments for drug delivery missed in a month in the center; (3) the cumulative proportion of women who suppressed viral load; and (4) the cumulative proportion of women with a suppressed viral load that has a rebound in viremia.

To evaluate the level of male involvement in the health of female partners (intermediate outcome), a dedicated questionnaire will be delivered to female partners at baseline (preintervention) and in the postintervention phase. The survey measures the following outcomes related to male involvement: (1) proportion of women accompanied by partners at the facility after receiving an invitation card; (2) proportion of men accepting HIV testing and counseling (if not yet received in the last 6 months); (3) level of partner involvement in the care process measured through a score scale based on health practices reported by the female partners; and (4) level of gender-based violence in the family measured through a score scale based on violent or negative practices reported by female partners.

Some indicators will be collected to monitor the delivery of the intervention and possibly evaluate correlations with the dose of delivery of the 3 interventions, as outlined in Textbox 1.

The primary and secondary indicators of effectiveness are the same for all interventions, hence making it possible to assess the differential impact of the community, health system, and structural-level interventions, particularly with respect to interventions based only on individual and interpersonal levels. These interventions will be compared with the usual services offered at the fourth center.

Data will be presented as mean (SD) for normally distributed data, median with IQR in case of nonnormally distributed variables, or percentage frequencies; within-patient comparisons will be made using an unpaired *t* test and chi-square test, as appropriate, at significance levels of *P*=.04. Statistical tests for the comparison of groups include the Mann-Whitney *U* and Kruskal-Wallis *H* tests for continuous variables, whereas the chi-square test will be used for categorical variables. Logistic regression models will be used to compare variables in bivariate and multivariate analyses. Relative risk with 95% CIs will be used to assess the strength of associations. Multivariate survival analysis evaluating retention in care as outcomes will be performed using Cox regression models.

**Indicators for each intervention.**

**Male champions (community level)**

- Number of meetings in the communities
- Duration of meetings
- Number of participants in each meeting
- Qualitative report of male champions on each meeting (qualitative data)

**Special day (health-system level)**

Number of men attending the special day
Basic data on men attending the special day:
  Employment status
  Family status
  Level of satisfaction of the intervention
  Number of partners of women accessing the health facilitys
Number of men receiving services offered during the special day (HIV test and counseling; blood pressure measurement; blood glucose; nutrition; health talks; drama)

**Nudge (structural level)**

Number of couples eligible for intervention
Number of incentives and counseling sessions delivered

## Results

The enrollment of patients in the preintervention phase started in July 2019 and ended on March 31, 2020. There is an ongoing analysis of preliminary data collected at the 4 centers during the preintervention phase. In total, 452 female participants were enrolled in the preintervention phase, with the following distribution: Namandanje (n=133); Kapeni (n=78); Kapire (n=75); and Balaka (n=166). Several meetings have been performed at the centers to organize the intervention phase. As of August 1, 2020, the interventions were initiated at the 3 centers and by September 2020 were ongoing.

## Discussion

### Study Strengths

This study aims to assess interventions targeted at enhancing the involvement of males in women antenatal health care in Malawi. Male involvement in prenatal care is still one of the lowest in the world in Malawi, ranging from 3.2% to 23% [27]. There are multiple reasons for the limited role of male partners in women’s health initiatives. Instead of addressing singular factors supposed to directly cause limited male involvement, the approach of our study is based on an ecological model [17]. The decision to base interventions on a theoretical framework is one of the strengths of this study. In fact, a theory-based approach allows us to better understand the reasons for failure or success of an intervention, develop better strategies, and evaluate their external validity [16,50]. In particular, the ecological model was chosen for this study because it considers not only the beneficiaries (HIV-positive women and their partners) and ART adherence as done in other studies [15] but also the social context that can facilitate or hinder the adoption of healthy practices, namely retention in care and adherence to therapy for HIV PMTCT. All the interventions being evaluated act on individual and interpersonal elements, through couple health education. In addition, each intervention addresses other elements such as the health structure, the community, or the structural level linked to transportation costs. This design allows us to understand how the different levels affect behaviors within the target group and, therefore, health outcomes. Another strength of these interventions is the implementation of these activities in a real-life setting, with the use of personnel already active in the center. This is a significant strength of this study, as it provides reproducible information to other sites.

### Limitations

The study design has some limitations. First, the nonrandomized assignment of the interventions based on feasibility criteria could be a possible selection bias. All patients were enrolled in a high-quality program delivered by an international nongovernmental organization, and the results could be different in other settings such as public health facilities. Second, the interventions are designed based on evaluations conducted in different countries and in a potentially different context. This poses the question of their applicability to the Malawian context. Third, the number of studies conducted on male involvement in Malawi is limited and, in many cases, outdated, which do not allow us to have current data at hand on male involvement in Malawi to compare with our ongoing analysis.

If the study is successful in identifying an effective intervention that enhances male involvement and reduces attrition of HIV-positive women in Malawi, then the resulting strategy can be implemented in many other sites in the country as well as in other countries. The results of the study will be shared with local and central health institutions in the country to disseminate our study findings and enhance good practices fostering improved outcomes for families living with HIV.

## Appendix

Multimedia Appendix 1 Guide on educational contents for staff involved in the project.

## Abbreviations

ANC: antenatal care
ART: antiretroviral therapy
DREAM: Disease Relief through Excellent and Advanced Means
MTCT: mother-to-child transmission
PMTCT: prevention of mother-to-child transmission
SSA: sub-Saharan Africa
