# Supplementary material for: Improving Treatment Adherence and Retention of HIV-Positive Women Through Behavioral Change Interventions Aimed at Their Male Partners: Protocol for a Prospective, Controlled Before-and-After Study
Source: JMIR Res Protoc. 2021 Jan 25;10(1):e19384. doi: 10.2196/19384 (PMC7870353; doi:10.2196/19384)
Supplement: Multimedia Appendix 1 [file resprot_v10i1e19384_app1.pdf]

Multimedia Appendix 1

Guide for healthcare staff involved in WEMEN project
How to become a better facilitator

This guide is useful tool for all the healthcare staff dealing with men involvement in WEMEN study. In fact, it gives you an overview of all the typical features of a successful facilitator and explain you how to become a better facilitator. Being a professional facilitator represents the key skills required for the good performance of the activities we are carrying out.

Introduction

It is not hard to create a good learning environment, and you do not have to be an expert to do it. But it is important to work on becoming a better facilitator by developing certain attitudes and skills.

This is especially important for men who are training to be Male Engagement (ME) facilitators. When men take on this facilitation role, they are not only being asked to help groups of men discuss issues of gender, violence, and sexual health. They are also being asked to model the attitudes and behaviours that men will need to protect their own and others' health, safety, and well-being.

Personal preparation

As a facilitator preparing to do ME work, you will need to look at your own thoughts and feelings and how these may affect your work. For example, you may feel uncomfortable talking openly about certain topics (for example, such aspects of sexuality). This will make it hard to facilitate a frank discussion. You may also have strong feelings about certain topics (for example, women carrying condoms). This may make it hard to facilitate an open discussion without imposing your own views. In doing this work, you may also be reminded of painful experiences from your own past, in which you suffered or caused others to suffer. Being reminded of these experiences may make it hard to talk about certain topics.

Active listening

Active listening is a basic skill for facilitating group discussions. It means helping people feel that they are being understood, as well as heard. Active listening helps people share their experiences, thoughts, and feelings more openly. It's a way of showing participants that their own ideas are valuable and important when it comes to solving their problems.

Active listening involves:

• Using body language to show interest and understanding. In most cultures, this will include nodding your head and turning your body to face the person who is speaking.
• Showing interest and understanding to reflect what is being said. It may include looking directly at the person who is speaking. In some communities, such direct eye contact may not be appropriate until the people speaking and listening have established some trust.
• Listening not only to what is said, but to how it is said, by paying attention to the speaker's body language.
• Asking questions of the person who is speaking, in order to show that you want to understand.
• Summing up the discussions to check that what has been said was understood. Ask for feedback.

Being nonjudgmental

Remember that information should be provided in nonauthoritarian, nonjudgmental, and neutral ways. You should never impose your feelings on the participants.

Effective questioning

Being able to ask effective questions is also a core skill for a ME facilitator. Effective questions help a facilitator to identify issues, get facts clear, and draw out differing views on an issue. Skillful effective questioning also challenges assumptions, shows you are really listening, and demonstrates that the opinions and knowledge of the group are valuable.

Effective questioning also increases participation in group discussions and encourages problem solving.

Ways to achieve effective questioning include:

- Ask open-ended questions: Why? What? When? Where? Who? How?
- Ask probing questions. Follow up with further questions that delve deeper into the issue or problem.
- Ask clarifying questions by re-wording a previous question.
- Discover personal points of view by asking how people feel and not just what they know.

Facilitating group discussions

There is no single best way to facilitate a group discussion. Different facilitators have different styles. Different groups have different needs. But there are some common aspects of good group facilitation, described below.

Setting the rules

It is important to create "ground rules" with which the group agrees to work. Ensure that ground rules are established regarding respect, listening, confidentiality, and participation.

Involving everyone

Helping all group members to take part in the discussion is a really important part of group facilitation. This involves paying attention to who is dominating discussions and who is not contributing. If a participant is quiet, try to involve them by asking them a direct question.

But remember that people have different reasons for being quiet. They may be thinking deeply! If a participant is very talkative, you can ask him/her to allow others to take part in the discussion and then ask the others to react to what that person is saying.

Encourage honesty and openness

Encourage participants to be honest and open. They should not be afraid to discuss sensitive issues. Encourage the participants to honestly express what they think and feel, rather than say what they think the facilitator(s) or other participants want to hear.

Keeping the group on track

It is important to help the group stay focused on the issues being discussed. If it seems as if the discussion is going off the subject, remind the group of the objectives for the activity and get them back on track.

Dealing with difficult people

People often take on certain roles within groups. Some of these roles can interfere with the learning of the workshop. Facilitating a group discussion may mean dealing with negative or disruptive people or someone who continues to interrupt the discussion. Reminding the group of the ground rules and asking everyone to be responsible for maintaining them is a good way to deal with difficult people.

If someone is always complaining, you can ask for specifics, address the complaint, or refer the complaint to the group. If a participant is disruptive, you can involve the group

by having its members ask the difficult person to help, rather than hinder, the group, or you can deal with him apart from the group.

Dealing with difficult situations

The ME manual addresses many topics that are very sensitive and difficult to discuss. The activities in WEMEN project create ways for these topics to be discussed openly in a group setting. But it is likely that ME facilitators will have to deal with participants who make statements that are not in line with the views and values of the program. These could include sexist, homophobic, or racist remarks or opinions. Everyone has a right to their opinion. But they do not have a right to oppress others with their views. For example, a participant might say, *"If a woman gets raped, it is because she asked for it. The man who raped her is not to blame."* It is important that ME facilitators challenge such opinions and offer a viewpoint that effects the philosophy of the program. This can be difficult. But it is essential in helping participants work toward positive change. The following process is one suggestion for dealing with such a situation:

Step 1: Ask for clarification

"I appreciate you sharing your opinion with us. Can you tell us why you feel that way?"

Step 2: Seek an alternative opinion

"Thank you. So at least one person feels that way, but others do not. What do the rest of you think? Who here has a different opinion?"

Step 3: If an alternative opinion is not offered, provide one

"I know that a lot of people completely disagree with that statement. Most men and women I know feel that the only person to blame for a rape is the rapist. Every individual has the responsibility to respect another person's right to say 'no.'"

Step 4: Offer facts that support a different point of view

"The facts are clear. The law states that every individual has a right to say no to sexual activity. Regardless of what a woman wears or does, she has a right not to be raped. The rapist is the only person to be blamed."

Please note that even after the facilitator takes these four steps to address the difficult statement, it is very unlikely that the participant will openly change his or her opinion. However, by challenging the statement, the facilitator has provided an alternative point of view that the participant will be more likely to consider and, it is hoped, adopt later.

Here are some general tips on presenting to groups:

- Practice any presentation beforehand.
- Move out from behind the podium or table and into the audience.
- Look at and listen to the person asking a question.
- Be aware of the sensitivities of your audience.

- Use humor, but do not wait for laughs.
- Never give a generic presentation. Try to customize it for the group, as there are many ways to cover the same material.

Educational material

1	*Women are more vulnerable to HIV/AIDS and other STI than men, so men are safe and there is no need to worry (circumcision)*	Men and women are equally vulnerable to sexually transmitted diseases including HIV. This means that if the man does not want to be sick, he must take care of his health. Moreover, in a couple, the health of one is linked to that of the other. So it is need to be healthy together	1) What is the difference between a man and a woman in term of getting HIV? 2) Do you think that women get infected by HIV more than men? 3) Do you know more HIV positive women than men? 4) Who has a shorter lifespan? 5) Who is more likely to die in	Men's and women's vulnerability to HIV and AIDS is largely determined by gender norms about sexuality. It is often assumed that "real men" have lots of sexual relations and that women should be coy and passive in sexual matters. As a result, women might not always have the power and/or skills to communicate and negotiate sexual behaviors and methods of prevention. Other factors, such as poverty, make it even less likely that men and women will be able to negotiate protection or even access important health information and services. Promoting women's rights to be free from discrimination, and

	road accidents? 6) Who is more likely to consume alcohol and get drunk? 7) Who is more likely to have sexually transmitted infections (STIs)? 8) Who is more likely to have more sexual partners and more unprotected sex? 9) Who is less likely to seek health	violence is an important step toward reducing vulnerability to HIV and AIDS. It is just as important to involve men in discussions about the role of negative gender norms and encourage them to discuss HIV and AIDS prevention with their partners.

			services?	
2	*Risky sexual behaviors make "**real men**".*	Often the idea of the "real man" is that of a **strong** man. And a strong man expose himself to risks without fear. Furthermore, a man who must appear strong can never be seen by other as weak, admit that he is	In your opinion, what does "real men" mean? How do a "strong man" behave? How does a man behave when he has health problems?	Many of men's risky sexual behaviors are rooted, to a large extent, in how boys and men are raised and socialized. These behaviors often put both men and young women at risk. Itis important for men to be aware of how gender norms influence decisions and behaviors and to think critically about the impact of those decisions and behaviors.

3	*A man who worries about his HIV status is not a real man.*	sick, and therefore ask a health worker for help. We must make it clear that this attitude does not reveal strength but fear. And that if you risk or don't ask for help, the result is the opposite: you get sick and you become weak. Being sick means not being able to work, not having fun, not being able to take care of		Gender rules may make it hard for men living with HIV and AIDS to ask for advice and support, since it might be seen as a sign of weakness. Men trying to live positively with HIV and AIDS face a range of challenges. They can range from the practical (such as losing a job) to the deeply emotional (feeling suicidal). They may be short-term crises or long-term conditions. Men may find it hardest to cope in areas that are most closely tied to their identities as men, such as sexuality. Sex, therefore, may be one of the most difficult issues with which men struggle. There are many possible resources

		your children and loved ones. So a true "real man" is one who does not risk, cares, and therefore can be a reference for his family. A STRONG man try to remain		men can use to help them live more positively with HIV and AIDS. They include talk to their wives, trust them and be more open. self-help groups, male friendly services, and role models of men who are living positively and openly with HIV and AIDS.
4	*Take care of their health makes men weak.*	strong and avoid any risky behaviors (not protected sex, misuse of alcohol, violent behaviours)		Men can use their privilege and power in several ways to prevent HIV/STIs and unintended pregnancies. The most immediate role men have in sexual health is in their own sexual lives. The privilege that men are granted because of their gender give them power over women in sexual decision

			making. With power comes responsibility. Men can use this responsibility to protect themselves and their sexual partners from HIV. But men also have power in the family, the community, and the workplace. They can use this power to promote HIV prevention, and support gender equality in order to reduce women’s sexual vulnerability.
5	*If you’re worried about your health, you’re weak.*		Most causes of death for men are associated with the self-destructive lifestyle many men follow. Around the world, they are pressured to act in certain ways. For example, men often take more risks, have more

				partners, and are more aggressive or violent in their interactions with others-all of which put them and their partners at risk. As men, it is important to be critical about your lifestyles and the ways you put yourselves at risk.
6	***Health is only a woman's problem*.**	It is not true that only women should be interested in health. First because by doing these men get sick and die before women (have you noticed that there are more widows		There is a clear relationship between how men are raised and if, and how, they worry about their health. Many men, as a way of showing their masculinity, do not worry about their health and may believe that taking care of the body or being overly concerned about health are female attributes. These kinds of attitudes and behaviors

than widowers?). Second because in a couple the health of one is linked to that of the other. So it is necessary that both of them are interested, otherwise the risk is that everyone in the family is ill.

are learned at early ages and impact men’s health throughout their lives.

7	***Women are considered less than men.***	Here it is a matter of reaffirming a basic human rights principle, namely that we are all human beings and therefore bearers of the same rights. Unfortunately, the idea that the strongest must prevail is very rooted and difficult to change. However, starting from the health issues it is possible to make people		Before starting a relationship, it is important to know and love yourself and to figure out what you might give in a relationship. Many single people think that they are ready for a relationship, but often they have not found out enough about themselves to truly be ready. As a result, they can sometimes find themselves in unhealthy relationships. Healthy relationships are about knowing and loving oneself first, and then identifying qualities about others that are of interest.
8	*Men are more knowledgeable than women*			

9	*Men's needs are more important than women.*	understand that we all get sick in the same way, we all die in the same way, and we can all contribute to the collective well-being in the same way. This is also why the woman must be respected and supported.		Healthy relationships are based on communication and mutual understanding and respect. Decisions are made together and neither person dominates the relationship. Unhealthy relationships, on the other hand, can mean poor communication and unequal decision making, which makes open talk about sexual behavior and contraception extremely difficult, and thus puts one or both partners at greater risk for STIs and HIV.
10	***Violence** is normal and*	In the face of misunderstanding	How do you face tension	Violence can be defined as the use of force (or the threat of

gives power over other person.	and anger, violence often seems the only possible reaction. Clearly the first point is that of women's rights, but it can also be added that violence is never the right way to solve any kind of problem. Usually after exerting violence on a woman the problems increase, and the anger does not	and conflict in his home life? What did it happen in case of disagreement with your partner? What characteristics make a relationship healthy? Why does violence usually occur in relationships? Are there differences between how	force) by one individual against another. Violence is often used as a way to control another person, to have power over them. It happens all around the world and often comes from the way individuals, especially men, are raised to deal with anger and conflict.

		fade. It is very important that other men, especially expert clients, communicate appropriately.	men and women express emotions? What are the differences?	
11	*It’s ok to use violence against women.*			Anger is a normal emotion that every human being feels at some point. The problem is that some people may confuse anger and violence, thinking they are the same thing and that violence is an acceptable way of expressing anger. However, there are many other ways of expressing anger, better and more positive ways. Learning to express our anger when we feel it is better than

			bottling it up inside. When we allow our anger to build up, we tend to explode.
12	*Violence makes a man stronger*.		Conflict happens in all relationships. It is the way that you handle this conflict that makes all the difference. Learning how to take the time to think about your feelings and express yourselves in a calm and peaceful way is an important part of building healthy and respectful relationships. As men, it is important to reflect on how you react when someone has a different opinion than yours or when someone does something that makes you angry. It is not always easy, but it is important

			to take the time to think about your feelings before you react, especially when you are frustrated or angry. Sometimes, if you do not take the time to think about your feelings, you may react in a way that is hurtful or violent to another person or even yourself. Men need skills and support to talk with their wives and girlfriends about creating healthier relationships.
13	*Violence is just physical, it can be also emotional*		
